# Phase separation enhanced magneto-electric coupling in La_0.7_Ca_0.3_MnO_3_/BaTiO_3_ ultra-thin films

**DOI:** 10.1038/srep17926

**Published:** 2015-12-09

**Authors:** A. Alberca, C. Munuera, J. Azpeitia, B. Kirby, N. M. Nemes, A. M. Perez-Muñoz, J. Tornos, F. J. Mompean, C. Leon, J. Santamaria, M. Garcia-Hernandez

**Affiliations:** 1 Instituto de Ciencia de Materiales de Madrid, Consejo Superior de Investigaciones Científicas, Sor Juana Inés de la Cruz, 3, ES-28049 Madrid, Spain; 2 Center for Neutron Research, National Institute of Standards and Technology, Gaithersburg, Maryland 20899, USA; 3 GFMC, Departamento de Física Aplicada III, Universidad Complutense, CEI Campus Moncloa, ES-28040 Madrid, Spain; 4 Laboratorio de Heteroestructuras con aplicación en Spintronica, Unidad Asociada Consejo Superior de Investigaciones Científicas/Universidad Complutense Madrid, Sor Juana Inés de la Cruz, 3, ES-28049 Madrid, Spain

## Abstract

We study the origin of the magnetoelectric coupling in manganite films on ferroelectric substrates. We find large magnetoelectric coupling in La_0.7_Ca_0.3_MnO_3_/BaTiO_3_ ultra-thin films in experiments based on the converse magnetoelectric effect. The magnetization changes by around 30–40% upon applying electric fields on the order of 1 kV/cm to the BaTiO_3_ substrate, corresponding to magnetoelectric coupling constants on the order of α = (2–5)·10^−7^ s/m. Magnetic anisotropy is also affected by the electric field induced strain, resulting in a considerable reduction of coercive fields. We compare the magnetoelectric effect in pre-poled and unpoled BaTiO_3_ substrates. Polarized neutron reflectometry reveals a two-layer behavior with a depressed magnetic layer of around 30 Å at the interface. Magnetic force microscopy (MFM) shows a granular magnetic structure of the La_0.7_Ca_0.3_MnO_3_. The magnetic granularity of the La_0.7_Ca_0.3_MnO_3_ film and the robust magnetoelastic coupling at the La_0.7_Ca_0.3_MnO_3_/BaTiO_3_ interface are at the origin of the large magnetoelectric coupling, which is enhanced by phase separation in the manganite.

The unusual phenomena appearing in materials with strongly correlated electrons are keenly studied, often with practical technological applications in mind^1^,^2^. In particular, the converse magnetoelectric effect (ME)^3^ observed in multiferroic materials that exhibit simultaneously ferroelectricity and magnetism opens a way to electrically control magnetization^4^,^5^. However, ferroelectricity and magnetism are unlikely to coexist, as the former breaks space-inversion and the latter time-inversion symmetry. Thus, intrinsic multiferroics are scarce^6^. An alternative towards magnetoelectricity is to combine ferroelectric (FE) and ferromagnetic (FM) materials in two-phase heterostructures^7^,^8^,^9^,^10^ usually in the form of composites or epitaxial thin films and multilayers^11^,^12^,^13^,^14^,^15^,^16^,^17^,^18^,^19^,^20^,^21^.

Two main coupling mechanisms between FE and FM order parameters have been used: strain mediated ME coupling^13^,^22^,^23^ and interfacial electronic reconstruction (charge coupling)^24^,^25^,^26^,^27^,^28^. A convenient way to quantify the magnetoelectric coupling is to represent the magnetisation changes induced by applied voltage with a linear magnetoelectic coupling constant:


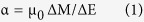


where ΔM is the change in the magnetization along the direction of the applied magnetic field and ΔE is the applied electric field perpendicular to the FM/FE interface^9^,^11^. The use of a simple α masks the drastic and non-linear changes induced by the ferroelectric coercive field. Effectively, it gives a lower limit to the ME effect. ME coupling constants as high as 2 ·10^−7^ s/m have been reported^11^ for strain mediated ME heterostructures. This generally acts through magnetostriction or piezomagnetism in the FM material^11^,^29^. However, in systems where interfacial electronic reconstruction is the main mechanism, theoretical calculations predict a change of the magnetic order, typically, in the first 3 unit cells of the manganite from FM to antiferromagnetic (AF)^24^,^25^, while several experimental works have obtained smaller ME coupling constants^30^,^31^.

In this *Report* we show strong magnetoelectric coupling found in a heterostructure of optimally doped FM La_0.7_Ca_0.3_MnO_3_ (LCMO, T_c_ ≈190 K) epitaxial ultra-thin (100 Å) film grown on BaTiO_3_ (BTO) as a FE substrate. This manganite is particularly prone to phase separation^32^ and, therefore, susceptible to being strongly affected by substrate strain and FE polarization. On the other hand, BTO is one of the most studied FE materials of the perovskite class^33^,^34^ and archetypal in the study of numerous FE phenomena, such as enhanced piezoelectric effects^35^. BTO undergoes three structural phase transitions: it is cubic above 393 K, tetragonal (T) down to 279 K (a = 3.994 Å and c = 4.036 Å), orthorhombic (O) down to 183 K (equivalent pseudo-monoclinic cell constant, c_m_ = 4.018 Å) and rhombohedral (R) at lower temperatures (a_R_ = 4.004 Å, α_R_ = 89.8°), remaining FE in all three lower temperature phases^33^. Recently, structural and electronic temperature dependent phenomena related to the chirality of the FE domain walls within the R phase have been predicted, suggesting a phase transition behavior around 100 K^36^. This is precisely the temperature range where LCMO/BTO heterostructures have been extensively studied; they exhibit exotic Matteucci magnetic loops, magnetic granularity^37^, and the coexistence of FM and AF phases^38^ originating from the magnetoelastic coupling between substrate and thin film^39^. In this system, the lattice mismatch between BTO and LCMO bulk parameters is large: η = (a_s_ − a_f_)/a_f_ ≈1–3%, where a_s_ and a_f_ are the substrate and thin film in-plane lattice parameters, respectively. This and the corrugation of the ferroelectric substrate strongly affect the magnetic and electronic transport properties of the manganite. Here we propose that the strain mediated magnetoelectric coupling in LCMO/BTO acts through phase separation by changing the fine grained domain structure of the manganite film and by inducing transitions to local antiferromagnetic order.

## Results and Discussion

In our experiments, we investigate the magnetoelectric effect in two different types of BTO substrates characterized by the presence of either only “c”-type FE domains, withpolarization pointing only along the normal to the sample surface, at room temperature (called pre-poled substrates throughout this paper), or having both “a”- and “c”-type domains within-plane and out-of-plane polarizations, respectively, equally present (called unpoled BTO substrates here). For further details see the Methods section.

### Magnetoelectric effect: Pre-poled substrates

**Figure 1:**
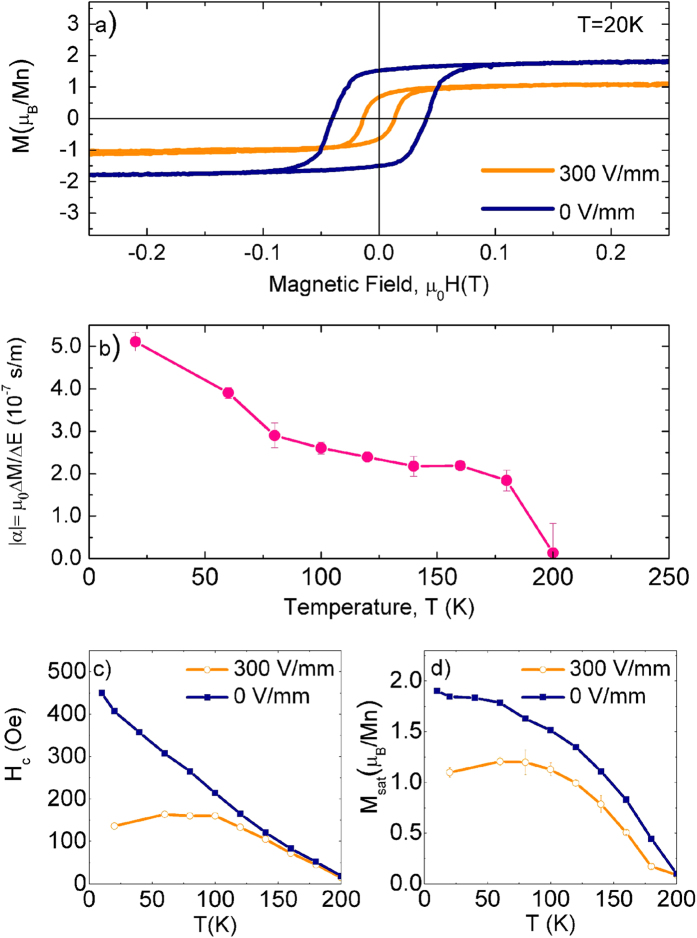
For a 100 Å thick LCMO film grown on a pre-poled BTO substrate, (**a**) Magnetic hysteresis loops at 20 K with no applied electric field (initial ferroelectric depolarized state, blue) and 3 kV/cm (applied electric field perpendicular to the thin film, orange). (**b**) Magnetoelectric coupling constant calculated from experimental values of ∆M and ∆E using Eq. (1). ΔM is the difference between saturation moments with no applied electric fields and a field of 3 kV/cm. Since the magnetic moment decreases for both positive and negative voltages with respect to the zero-voltage state, we do not assign a sign to the ME constant |α|. (**c**,**d**) coercive fields and saturation moments vs. temperature, respectively, extracted from magnetic hysteresis loops. Notes: 1 Oe = 79.577 A/m, the confidence interval for the error bars is one standard deviation.

Figure 1a shows the magnetic hysteresis loops of LCMO/BTO (pre-poled substrate) measured at 20 K without and with an applied electric field of 3 kV/cm, after correcting for the substrate diamagnetism. Two remarkable differences can be observed upon electric field application: i) strong reduction (38%) of the saturation moment, decreasing from M_sat_ ≈1.9 μ_B_/Mn to M_sat_ ≈1.1 μ_B_/Mn and, ii) reduction of the coercive fields, from 450 Oe to 150 Oe. From the temperature evolution of the change in saturation moment, ΔM(T)=M(T,0kV/cm)–M(T,3 kV/cm), in the ferromagnetic phase of the LCMO and in the R phase of the BTO substrate, we can calculate the linear magnetoelectric coupling constant as a function of temperature using Eq. (1), resulting, at low temperatures, in a maximum absolute value of |α| = 5·10^−7^ s/m, as depicted in Fig. 1b. Our estimation for the magnetoelectric constant is the result of comparing two states of the LCMO that arise from the different average strains imposed by the BTO substrate, when brought to the R phase with and without applied electric field.

The ME effect was observed at all temperatures in the FM phase of the LCMO. In Fig. 1c,d, we compare the coercive fields and saturation moments in the ferroelectric depolarized (“c” domains pointing both towards and against the LCMO thin film are expected, in blue) and polarized (“c” domains all pointing in the same direction, in orange) states of the BTO. The effect of electric polarization of the substrate is striking: the magnetization is strongly reduced and this reduction is stronger at lower temperatures. Furthermore, the magnetization reaches a broad maximum in the polarized state between 50 and 100 K, as opposed to the monotonic behavior in the depolarized one. The coercive field also ceases to grow linearly with decreasing temperature when applying electric fields, and instead becomes practically constant below 100 K. This suggests a change of magnetic anisotropy (reflected by the change in the shape of magnetic hysteresis loops) due to changes in the ferroelectric substrate and implies a modification in the magnetic granularity of the system below 100 K^37^,^40^.

The main mechanisms allowing for magnetoelectric coupling in this type of heterostructure are interfacial electronic reconstruction and strain. To discern between these mechanisms we look at the “symmetric” nature of the magnetoelectric effect in LCMO/BTO: we observed exactly the same behavior when polarizing the substrate with polarization vector pointing towards the thin film or away from it. Thus, the magnetoelectric coupling observed in these LCMO/BTO samples must be largely due to a strain mediated mechanism, instead of interface reconstruction, where differences with different directions of the polarization are expected^24^,^25^,^26^.

### Magnetoelectric effect: Unpoled substrates

**Figure 2:**
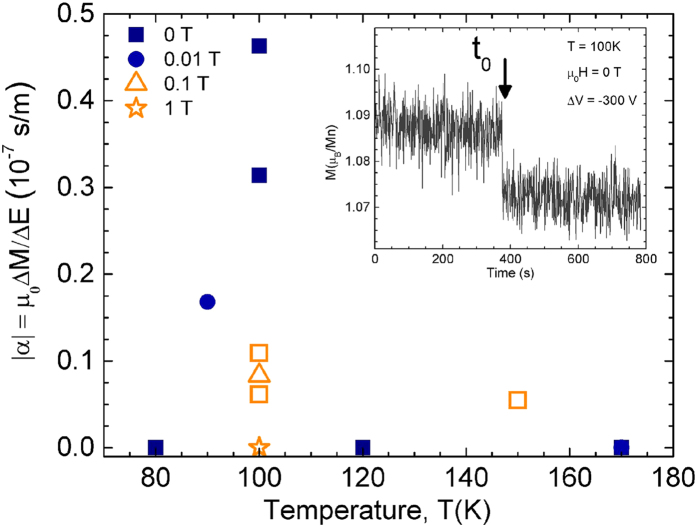
Magnetoelectric coupling constant, calculated from experimental values of ∆M and ∆E according to Eq. (1), plotted as a function of temperature for two representative LCMO/BTO samples grown on unpoled substrates (in orange and blue). Different shapes correspond to different applied magnetic fields, as indicated. Inset: Magnetic moment vs. time, and in magnetic remanence after field cooling in 0.1 T. The black arrow indicates the time, t_0_, in which an electric field of 3 kV/cm (pointing away from the thin film) is applied to the ferroelectric substrate resulting in a decrease in magnetization (magnetoelectric effect).

LCMO thin films grown on unpoled BTO substrates behave differently. Although a magnetoelectric effect is present, it is one order of magnitude weaker (|α|≈5·10^−8^ s/m). Figure 2 shows α values as a function of temperature measured on two representative samples, in various magnetic fields (as indicated).

A remarkable difference compared with the samples grown on pre-poled substrates is the dependence of α with temperature and applied magnetic fields: no magnetoelectric effect was observed in these samples at temperatures below 80 K or at magnetic fields higher than 0.1 T (lower detection limit for |α| in our experimental setup with the magnetic moments of these samples is 10^−9^ s/m). It was, in fact, impossible to observe magnetoelectric coupling while measuring magnetic hysteresis loops or magnetization vs. temperature curves (in contrast to thin films grown on pre-poled substrates), and the procedure reported in ref. 11 was followed instead. First, the sample was cooled in a magnetic field below coercivity (or even in no magnetic field). Then, while monitoring the magnetic moment at a certain time, t_0_, an electric field larger than the switching field of the substrate (1–3 kV/cm) was applied in a step-like fashion, causing corresponding step-change in the observed magnetization. This was always well-defined, did not decay over time and implied a reduction of the magnetization (see example in the inset to Fig. 2).

In addition, the change in magnetic moment brought on by the applied electric field was only observed for a limited number of “switching” events, as the successive ΔM values quickly decreased when consecutively applying electric fields, until no effect remained. Nevertheless, the effect was persistent in time and largely reversible in the sense that removing the electric field resulted in a recovery of the initial magnetic moment. Several methods to recover the initial state of the sample, and therefore, the possibility of observing the magnetoelectric effect again, were investigated (as, for example, depolarizing electrically and magnetically the samples at room temperature or heating the samples over the ferroelectric Curie temperature of BTO in O_2_ atmosphere), to no avail.

### Magnetization profiles

PNR can reveal the depth profile of the magnetization component perpendicular to the neutron momentum transfer, Q, within thin films and multilayers^41^,^42^. Layer thicknesses in the sample are manifested in the wavelengths of oscillations in the Q-dependent reflectivity. Thus, real space resolution is determined in part by the maximum value of the measured Q range. For this work, we were unable to resolve a full oscillation corresponding to the LCMO film thickness, hindering the quantitative characterization of the structure or magnetic profile.

**Figure 3:**
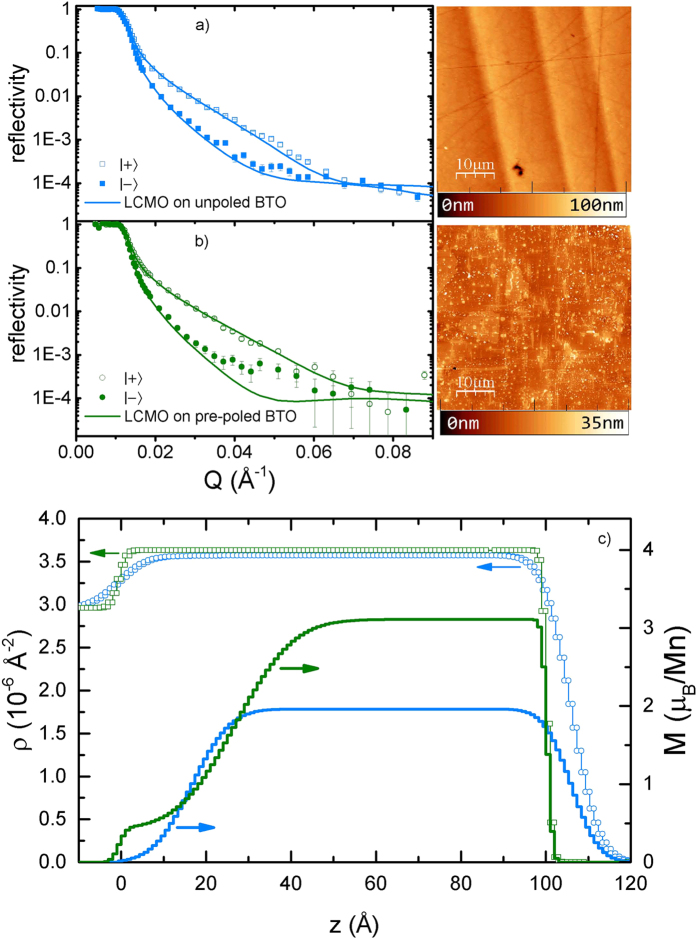
Upper panels show PNR data (symbols) and fittings (lines) measured at 30 K and after field cooling in 0.8 T, and the corresponding AFM topography images at room temperature, for two samples grown on unpoled (**a**) and pre-poled (**b**) substrates. (**c**) Structural (symbols) and magnetic (lines) profiles for the same samples. The confidence interval for the error bars is one standard deviation.

Figure 3 shows, at 30 K, reflectivities for samples grown on un- and pre-poled BTO substrates. With the thickness of these samples (100 Å) and wavelength of the neutron beam (4.74 Å), only half of one broad oscillation can be appreciated in the reflectivity curves in the Q range from 0 to 0.08 Å^−1^.

PNR magnetization and nuclear scattering length density profiles have been successfully obtained for our LCMO thin films before^39^. Here, we show similar results for another sample. For the Polarized Beam Reflectometer instrument at NIST (PBR)^43^, the neutron coherence length is greater than 1 μm^44^. For the model fitting, we assume that domains in the sample are small enough with respect to the neutron coherence length. This way the scattering length density is the result of the average scattering potentials of multiple domains (as opposed to coherent scattering from multiple domains). Further, we assume a specific model in which the LCMO layer was partitioned into two different sub-layers (depleted and bulk-like LCMO sub-layers). Non spin-flip reflectivities and the corresponding best fittings are shown in Fig. 3a. Spin-flip reflectivities were undetectable with our instrumental setup. The resulting profiles (Fig. 3c, in blue) are similar to those obtained in Ref. 39 showing overall depressed magnetization along the profile with even lower values near the BTO substrate. Roughness is relatively small and, in agreement with atomic force microscopy topography (AFM), has an average value of approximately 10 Å. The lack of sharpness of the interfaces, as already discussed in Ref. 39, is the result of the BTO corrugation and domain misalignment (up to 1°), which is transmitted from the BTO/LCMO interface to the LCMO free surface^38^.

The case of thin films grown on pre-poled BTO substrates is very similar to those on unpoled substrates, and the reflectivities for the two samples exhibit equivalent trends (Fig. 3b). Nevertheless, two differences can be pointed out: first, the overall intensity of the reflectivity for the LCMO on un-poled substrate decays slightly faster in all the measurable Q range than for the pre-poled sample, which we attribute to the effect of corrugation. Second, at low Q values, the difference between the two non-spin flip reflectivity curves is larger in the pre-poled sample than in the unpoled one, indicating differences in the distribution of magnetization across the profile. These qualitative observations will show up as differences in the magnetic depth profiles obtained in our fittings.

The fittings of the sample grown on the pre-poled BTO substrate were done using exactly the same model that was used for the unpoled sample. The nuclear scattering length profiles show relatively flat interfaces (no corrugation). The typical roughness is of 3–5 Å. This is in accordance with those measured at room temperature with AFM, (see right panel in Fig. 3b), and consistent with the slower decay of the reflectivity curves.

The lack of corrugation in samples on pre-poled substrates allows us to better characterize the manganite magnetic profile. Since the profiles are not affected by the “smoothing” effect of misaligned FE domains (see Fig. 3c) the uneven distribution of magnetization across the depth profile is apparent. On our LCMO/BTO samples grown on pre-poled substrates we are able to see for the first time a profile with non-zero, but strongly reduced, magnetization near the interface. This seems to be a general trend on the fittings performed with this model. However, the thickness and magnetization of the depleted layer are coupled values. That is, very similar results/fittings can be obtained by increasing the depleted layer thickness together with the magnetization near the interface. This also appears in the fitting as a high roughness between the depleted layer and the bulk-like side of the LCMO thin film (of about 20 Å in our fitting), indicating that the magnetic profile is not step-like but rather the magnetization increases as we move away from the interface.

We emphasize, nonetheless, that the high similitude between reflectivity curves in the two samples and the absence of more features in the available Q range make very difficult to extract quantitative data from these reflectivity curves, and, therefore, we concentrate on the qualitative main features of these reflectivities: 1) the effect of RT FE domains of the substrate and, 2) the non-uniform distribution of the magnetization across the profile. Despite the similitudes between reflectivity curves, the average magnetizations calculated from the fittings coincide with average saturation moments obtained for these samples in VSM measurements: 226 kA/m (1.42 μ_B_/Mn) for LCMO grown on unpoled BTO and 328 kA/m (2.06 μ_B_/Mn) on pre-poled BTO.

### Magnetic domain maps

**Figure 4:**
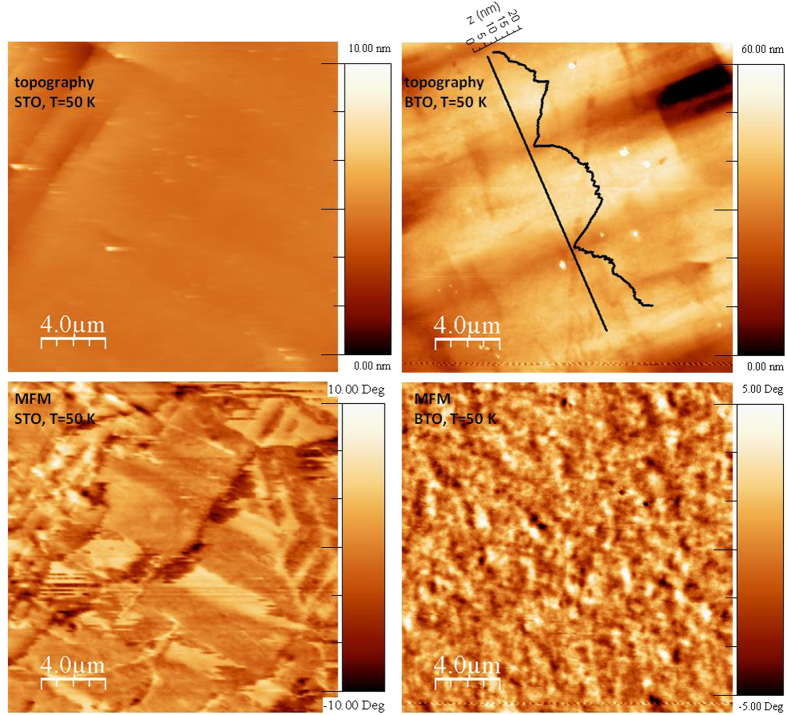
AFM topography and MFM images of LCMO/STO and LCMO/BTO films at 50 K. In the LCMO/BTO topography images the line-cuts indicate the large FE a- and c-type domains of the unpoled BTO.

Low temperature magnetic force microscopy (MFM) shows that the magnetic domain structure of LCMO on BTO consists of small grains with typical sizes around 300 nm, with an isotropic distribution (Fig. 4). This magnetic granularity is decoupled from the topographic features, which vary on the scale of several microns, with a well-defined directionality. It is also in stark contrast to the well-defined magnetic domains of LCMO on SrTiO_3_, which extend over several microns.

### Mechanism for LCMO/BTO Magnetoelectric Coupling

A dominant mechanism based on interfacial electronic reconstruction^24^ is ruled out by the observed symmetric ME response for positive and negative applied voltages. However, the large ME effect seen in LCMO on pre-poled BTO cannot be caused solely by magnetoelasticity, either. To see why, we consider for a moment such a strain-mediated ME effect, assuming perfect coupling between the FM and FE layers in a bilayer heterostructure. We can then estimate the order of magnitude of the magnetoelectric coefficient, α, from:


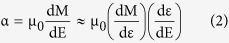


where M is the magnetization, E the applied electric field and ε represents the strain at the FE/FM interface. The term dm/dε can be approximated from a magnetoelastic model of the LCMO layer, while the piezoelectric coefficients for BTO provide an estimate for dε/dE. An order of magnitude estimate for the magnetoelastic derivative comes from the product of a geometric term (which we evaluate under the assumption of equilibrium, cubic symmetry and no shear stress) involving only the direction cosines of the magnetization vector, **m**, and the saturation magnetization in the direction of the applied field, giving:


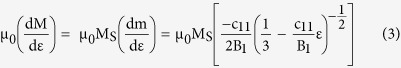


with 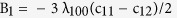
where c_11_ = 350 GPa, and c_12_ = 113 GPa are elastic constants and λ_100_ = 7·10^−5^ is the magnetostriction of LCMO^45^,^46^. The order of magnitude of the strain is ε = 1%. With M_S_ measured in the range of 1–2 μ_B_/Mn, we estimate 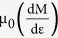 to be on the order of 100 T. For the piezoelectric derivative we take 85 pm/V for BTO^33^. Thus, magnetoelastic coupling does not account for more than α around 10^−8^ s/m, an order of magnitude less than that observed in LCMO on pre-poled substrates and can only explain the ME effect observed in samples grown on unpoled substrates.

The key to understand the ME mechanism in LCMO/BTO is the depleted magnetic layer, observed for pre-poled LCMO/BTO in Fig. 3c, which originates from the heterogeneous and large lattice mismatch between the BTO substrate and LCMO thin film. Again, since the magnetization is equally diminished for positive and negative variations of the electric field, effects due to oxygen reorganization in the LCMO thin film or depletion/accumulation of charges in the BTO side of the interface are ruled out.

**Figure 5:**
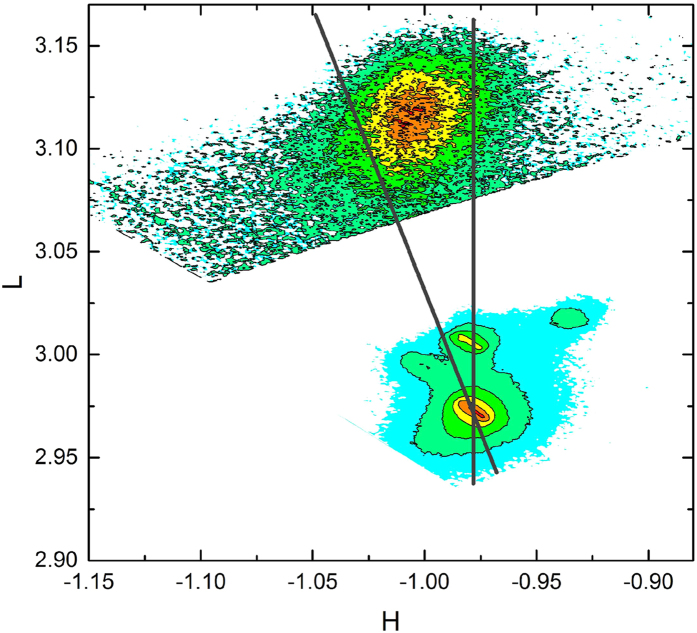
Reciprocal space map of LCMO thin film grown on a pre-poled BTO at room temperature around the (−103) peak, as indexed to BTO. The broadening along the H direction is consistent with considerable variations of the LCMO in-plane lattice parameter. Straight lines correspond to fully relaxed and fully epitaxial LCMO. Inset: Model of the in-plane strain distribution along the sample plane with and without electric field applied to the substrate, around the nominal strain value.

Contrary to what would be expected for a homogeneously strained film, the reduction of the magnetization occurs when the strain of the LCMO film is relieved as electric fields are applied to the BTO (in analogy with Ref. 11). The in-plane lattice parameter is expected to fluctuate laterally, along the substrate, while at the same time relaxing away from it. LCMO is prone to phase separation and such strain variations along the LCMO/BTO interface disturb the equilibrium between phases, allowing the coexistence of varying ratios of FM and AF phases in the LCMO thin film^37^,^38^,^39^. This situation has been previously observed in LCMO grown under high compressive strain in refs. 47,48, so it is plausible to assume a similar situation for large tensile strain, such as our case. Figure 5 depicts the reciprocal space map for the (−103) reflection of a LCMO/BTO sample on a pre-poled substrate. The broadening along the in-plane direction is consistent with just such a dispersion of biaxial in-plane strain (as well as with the, usually considered, finite size coherence effects), a possible strain relief mechanism^49^,^50^ distinct from coherent epitaxy.

Crucially, the applied electric field lowers the BTO in-plane lattice parameters, causing the average LCMO strain to decrease. Thus, the overall strain distribution is also lowered, increasing the expected concentration of AF clusters near the interface through phase separation^51^ and lowering the overall magnetization (Fig. 1a). The phase separated LCMO is then more homogeneously distributed laterally, being AF near the interface and FM further away. However, it also leads to less intimate coupling to, and so less granular magnetic domains in, the FM over layer. The magnetic anisotropy in LCMO/BTO is intimately linked with the magnetic granularity^39^, so in the FE polarized state our model predicts less anisotropic magnetism. Indeed, Fig. 1 shows very different behavior of the manganite film with and without applied electric field. The blocking temperature is reduced and the coercive field does not decrease linearly in the polarized state. Both indicate reduced magnetic anisotropy. The LCMO on FE polarized BTO behaves like a less strained, less granular, more homogeneously ferromagnetic, but effectively thinner, FM LCMO film.

## Conclusions

In summary, we have demonstrated very large magneto-electric coupling in ultrathin manganite (LCMO) films grown on pre-poled ferroelectric BTO substrates. The ME coupling was shown to be the same for positive or negative electric fields, thus excluding a dominant role of interface reconstruction based coupling mechanisms. We have shown that conventional magneto-elastic coupling alone cannot account for the observed absolute value of α ~5·10^−7^ s/m ME coupling. Polarized neutron reflectometry revealed a well-defined depressed magnetic layer at the interface under an overall better magnetized layer. Magnetic force microscopy revealed complex, fine-grained magnetic domain maps in LCMO/BTO. Magnetometry revealed that the magnetically granular behavior changes upon applying an electric field, to a less granular state. We propose that phase separation enhances the magneto-electric coupling in LCMO thin films on BTO.

## Methods

LCMO epitaxial thin films were grown simultaneously on nominally [001] oriented BTO and [100] oriented STO single crystal substrates using high O_2_ pressure (3.4 mbar) dc sputtering at 900 °C. We performed ME experiments in samples grown on two different types of BTO substrates, both acquired from MaTecK: cut from unpoled and poled crystals. Unpoled substrates exhibit, at room temperature (BTO T phase), both “a”-type (FE polarization lies in-plane) and “c”-type (FE polarization points out-of-plane) FE domains; pre-poled substrates were sorted by X-ray diffraction at room temperature and only those exhibiting mostly “c”-type FE domains (with c/a domain ratio higher than 90%) were chosen.

Magnetic characterization and measurements of the converse magnetoelectric effect were performed using a Quantum Design VSM-PPMS system. For the simultaneous application of external voltages to the samples while magnetic measurements were in progress, we used a modified sample holder and sample rod equipped with two wires of Manganin® alloy. Voltage differences up to ±5 kV/cm were applied between the top electrode formed by the LCMO layer and a bottom electrode (silver paint on the unpolished BTO side) using a Keithley 2410 SourceMeter. To avoid arcing, the PPMS sample chamber was purged with He and kept at high vacuum.

The ME effect was best observed when adopting the following protocol: in the ferroelectric tetragonal phase (T = 300 K) an electric field of the desired intensity (typically ± 3 kV/cm), higher than the ferroelectric coercivity, was applied normal to the sample surface. Direct observation of the current peak at the switching field (using the SourceMeter) ensured that the sample was properly contacted and that the polarization switching process was taking place. The electric field was then maintained during all subsequent measurements at lower temperatures, to maintain the direction of the polarization of the BTO.

Profiles of the in-plane component of the magnetization in the LCMO layer were determined by polarized neutron reflectometry (PNR) performed at the PBR Polarized Beam Reflectometer (NIST Center for Neutron Research)^43^ for 100 Å thick LCMO films grown on a 5 × 5 × 1 mm^3^ pre-poled and a 10 × 10 × 1 mm^3^ unpoled BTO substrate. In PNR experiments, the sample was cooled to 30 K under a 0.8 T saturating magnetic field applied in-plane, along the BTO [110] direction, coincident with the LCMO magnetic easy axis. Magnetization and nuclear scattering length profiles were extracted by least squares fittings to models using the Refl1d software suite^52^.

Certain commercial equipment, instruments, materials, suppliers, or software, etc., are identified in this paper to foster understanding. Such identification does not imply recommendation or endorsement by the National Institute of Standards and Technology, nor does it imply that the materials or equipment identified are necessarily the best available for the purpose.

## Additional Information

**How to cite this article**: Alberca, A. *et al.* Phase separation enhanced magneto-electric coupling in La_0.7_Ca_0.3_MnO_3_/BaTiO_3_ ultra-thin films. *Sci. Rep.* **5**, 17926; doi: 10.1038/srep17926 (2015).
